# Complete mitochondrial genome of the marine polychaete, *Nereis zonata* (Phyllodocida, Nereididae) isolated from the Beaufort Sea

**DOI:** 10.1080/23802359.2020.1861994

**Published:** 2021-01-19

**Authors:** Sang-Eun Nam, Somyeong Lee, Tae-Yoon S. Park, Jae-Sung Rhee

**Affiliations:** a Department of Marine Science, College of Natural Sciences, Incheon National University, Incheon, South Korea; b Division of Polar Earth-System Sciences, Korea Polar Research Institute, Incheon, South Korea; c Polar Science, University of Science & Technology, Daejeon, South Korea; d Research Institute of Basic Sciences, Incheon National University, Incheon, South Korea

**Keywords:** Beaufort Sea, mitogenome, Nereididae, Nereis zonata, polychaete

## Abstract

Here, we present the first whole mitogenome sequence of the marine polychaete, *Nereis zonata*, isolated from the Beaufort Sea. The mitochondrial genome of *N. zonata* is 15,757 bp in length and consists of 13 protein-coding genes (PCGs), 22 transfer RNA (tRNA) genes, 2 ribosomal RNA (rRNA) genes, and a non-coding region that is typical of polychaetes. GC content of the *N. zonata* mitogenome is 37.2%. A maximum-likelihood gene tree based on the *N. zonata* mitogenome combined with previously published annelid mitogenome data revealed that *N. zonata* is clustered with *Cheilonereis cyclurus*, which form a sister group to *Nereis* sp.

Annelids are the ringed or segmented worms, which are found in a wide range of terrestrial, freshwater, and marine habitats with complex ecological and morphological diversity (Rouse and Fauchald 1995; Struck et al. 2011). Phylogeny within the group Annelida remains undetermined; however, monophylogenetic analysis suggests the existence of three main clades, including Sipuncula, Errantia, and Sedentaria (Andrade et al. 2015). Among these, Errantia comprises Aciculata (Phyllodocida + Eunicida) and Protodriliformia (Weigert and Bleidorn 2016). The aciculatan family Nereididae (Weigert et al. 2014) comprises at least 540 species and 43 genera (Bakken and Wilson 2005). *Nereis zonata* Malmgren, 1867 (Phyllodocida, Nereididae) exhibits a wide geographical distribution, ranging from the Indo-Pacific to the Northeast Atlantic and the Arctic oceans. This typical Arctic-boreal species have been reported from different regions of West to East Greenland (e.g., Spitsbergen, Novaya Zemlya, and the Murman coast) (Wesenberg-Lund 1951). However, genomic information on *Nereis zonata* is limited, as only two marker genes, including *COI* and *28S rRNA*, have been registered at NCBI GenBank.

An individual organism of *N. zonata* was isolated from the Beaufort Sea (69°52′N, 139°03′W) in 2017 using a remotely operated underwater vehicle (ROV) belonging to the Monterey Bay Aquarium Research Institute (MBARI). The specimen was deposited in the Korea Polar Research Institute (Species ID: Annelid-02; Specimen ID: KOPRI-Benthos-08). Genomic DNA was extracted from the muscle tissue of the specimen using a DNeasy Blood and Tissue kit (Qiagen, Hilden, Germany) according to the manufacturer’s instructions. The *COI*, *cob*, and *rrnL* genes were amplified by PCR with the universal primers and degenerative primers (Folmer et al. 1994). By several combinations of additional primers targeting the gaps between partial genes, long fragments were amplified using a long-PCR technique. Detailed experimental conditions for the long-PCR is followed: 40 cycles of 98 °C for 25 s and 68 °C for 12 min in a 50 μL reaction mixture containing 30.5 μL distilled water, 5 μL 10 × LA PCR buffer II (TaKaRa, Japan), 8 μL dNTP (4 mM), 5 μL of each primer (5 μM), 0.5 μL LA Taq polymerase (2.5 U), and 1 μL of *N. zonata* genomic DNA. To obtain a circular complete mitogenome, the long fragments were sequenced directly using a genome-walking PCR technique at Bionics (Seoul, South Korea). Additional PCR procedures and sequencing were conducted to confirm the entire mitogenome. The resulting circular sequence was annotated using MITOS2 (Bernt et al. 2013) and tRNAscan-SE 2.0 (Lowe and Eddy 1997) and confirmed using NCBI-BLAST (http://blast.ncbi.nlm.nih.gov).

The complete circular mitogenome of *N. zonata* was 15,757 bp in length (GenBank Accession no. MT980928), containing 13 PCGs, 22 tRNAs, two rRNAs, and one non-coding conserved region. The percentages of A, T, C, and G were 31.5%, 31.3%, 15.6%, and 21.6%, respectively. The nucleotide composition was significantly biased toward A + T nucleotides. The *COI* sequence was identical to the five partial *COI* sequences of *N. zonata* that were previously submitted (577-600 bp; GenBank accession no. HQ024401-HQ024405) (Carr et al. 2011). The overall genome architecture of the *N. zonata* mitogenome is conserved, and similar to other mitogenome sequences of the Nereididae. Phylogenetic relationships were predicted using the concatenated set of the whole 13 PCGs of the *N. zonata* mitogenome, 12 published mitogenomes belonging to Nereididae, and 13 registered mitogenomes of other annelid species (Park et al. 2020) (Figure 1). Overall, the relationship between major clades followed a well-established annelid phylogeny (Weigert and Bleidorn 2016), as Errantia is separated from Sedentaria. Families belonging to Errantia also exhibit reliable phylogenetic relationships suggesting that Nereididae is closely related to Syllidae and Glyceridae, thereby forming a cluster with Goniadidae (Weigert and Bleidorn 2016; Chen et al. 2020). Further information on mitogenomes of the family Eunicidae would be helpful in establishing a robust molecular phylogeny. The *N. zonata* mitogenome was closely clustered with *Cheilonereis cyclurus*, thereby forming a sister group of *Nereis* sp., which was fully supported with a high bootstrap value. Based on a previous morphological characteristics-based phylogenetic analysis, *C. cyclurus* was placed in a clade together with *Perinereis* and *Pseudonereis* species, while *Nereis* species were grouped in another clade, thereby forming *Neanthes* along with several *Perinereis* species (Bakken and Wilson 2005). To the best of our knowledge, our study is the first to report the close relationship between *Nereis* and *Cheilonereis*. Altogether, the complete mitogenome sequence of *N. zonata* serves as a robust resource for understanding the phylogenetic relationship of *Nereis* species and the evolutionary history within the family Nereididae.

**Figure 1.**
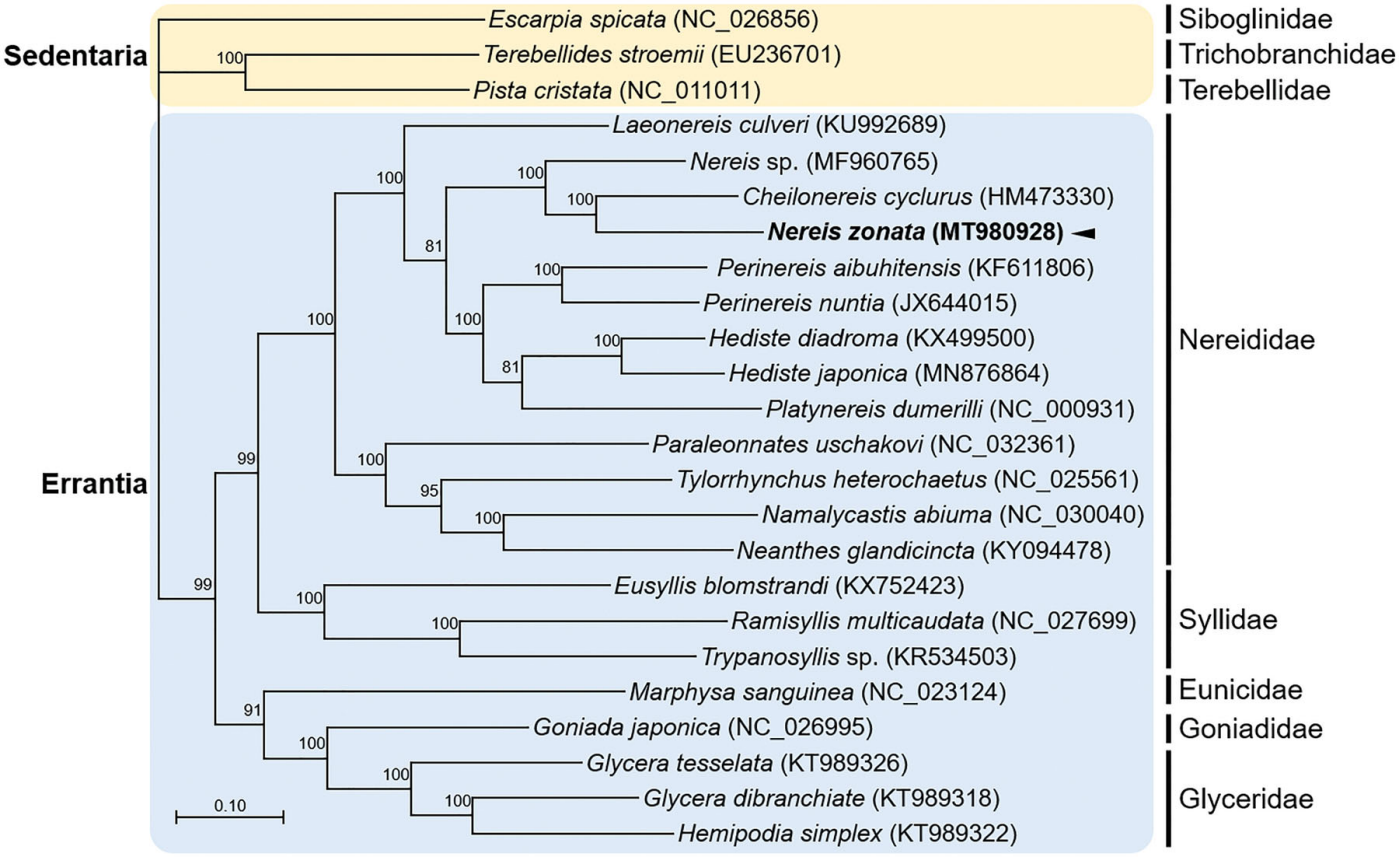
Maximum-likelihood (ML) phylogeny of 13 published mitogenomes from Nereididae including *N. zonata* and 13 registered mitogenomes of other annelid species based on the concatenated nucleotide sequences of protein-coding genes (PCGs). The phylogenetic analysis was performed using the maximum likelihood method, GTR + G + I model with a bootstrap of 1000 replicates. Numbers on the branches indicate ML bootstrap percentages. DDBJ/EMBL/Genbank accession numbers for published sequences are incorporated. The black arrow means the marine polychaete analyzed in this study.

## Data Availability

The data that support the findings of this study are openly available in the National Center for Biotechnology Information (NCBI) at https://www.ncbi.nlm.nih.gov, accession number MT980928.
